# Dietary phytochemicals targeting neuroinflammation: mechanisms, bioavailability and translational challenges

**DOI:** 10.3389/fphar.2026.1943126

**Published:** 2026-10-26

**Authors:** Kumar M. R. Bhat, Raghavendra Upadhya, Aashika Nayak, Anjaneyulu Konuri, Praveen Kumar S. E., Murali Adiga, Vasanthalaxmi K Rao, Dinesh Upadhya

**Affiliations:** 1 Department of Anatomy, Kasturba Medical College, Manipal Academy of Higher Education, Manipal, India; 2 Department of Biotherapeutics Research, Manipal Academy of Higher Education, Manipal, India; 3 Department of Anatomy, Manipal Tata Medical College, Manipal Academy of Higher Education, Manipal, India; 4 Department of Pharmacology, Manipal Tata Medical College, Manipal Academy of Higher Education, Manipal, India; 5 Department of Physiology, Kasturba Medical College, Manipal Academy of Higher Education, Manipal, India

**Keywords:** animal models, anti-inflammatory drugs, cytokines, neuroinflammation, phytochemicals

## Abstract

Neuroinflammation and oxidative stress are among the most common and fundamental biochemical mechanisms underlying many neurological disorders, and both are promising therapeutic targets. Neuroinflammation and oxidative stress are bidirectional, and each could trigger and amplify the other. Since activated immune cells during neuroinflammation produce high levels of ROS, creating a vicious biochemical cycle, targeting neuroinflammation is an ideal opportunity to curtail both neuroinflammation and oxidative stress. The initiation and progression of neuroinflammation cause structural and functional damage to both neurons and glial cells in the central nervous system. Early intervention to inhibit neuroinflammation could prevent irreversible damage to the nervous tissue. The current application of glucocorticosteroids and nonsteroidal anti-inflammatory drugs in neurological disorders poses a risk of injury to the cardiovascular, gastrointestinal, respiratory, hepatic, and renal systems. Bioactive phytochemicals derived from dietary herbal medicines with demonstrated anti-inflammatory activity could serve as effective substitutes. Although very few of these phytochemicals have undergone clinical trials demonstrating efficacy and safety, several limitations, including lower bioavailability, solubility, and stability, hinder their clinical use. The purpose of this review is to identify preclinical data on multiple dietary bioactive phytochemicals with robust anti-neuroinflammatory effects that could be considered for clinical trials. Based on sufficient, efficacious, predominantly preclinical data, this review highlights the need for future modifications to enhance bioavailability while maintaining or improving therapeutic efficacy, with reduced systemic side effects, for these phytochemicals, rather than evaluating their potency in newer disease models or exploring new mechanisms. Further, prioritizing the identification of top-tier phytochemicals or their combinations that exhibit high bioavailability, optimal dosing, and proven long-term safety, while minimizing drug interactions and systemic toxicity through a standardized set of preclinical studies, is essential to manage chronic neuroinflammation with phytochemicals.

## 1 Introduction

In many central nervous system disorders, chronic neuroinflammation is either the etiology or a key factor in disease progression. The etiology of neuroinflammation may involve oxidative and non-oxidative injury, molecules released from damaged cells, toxins, or pathogens, and stimuli that trigger innate immune responses, which is known to be the earliest initiator of the inflammatory cascade. This process involves cell-surface receptors, signal transducers, activators of transcription, and inflammatory mediators (Zhang et al., 2023). Biochemical processes in neuroinflammation involving microglia and astrocytes in the brain are demonstrated in Figure 1.

**FIGURE 1:**
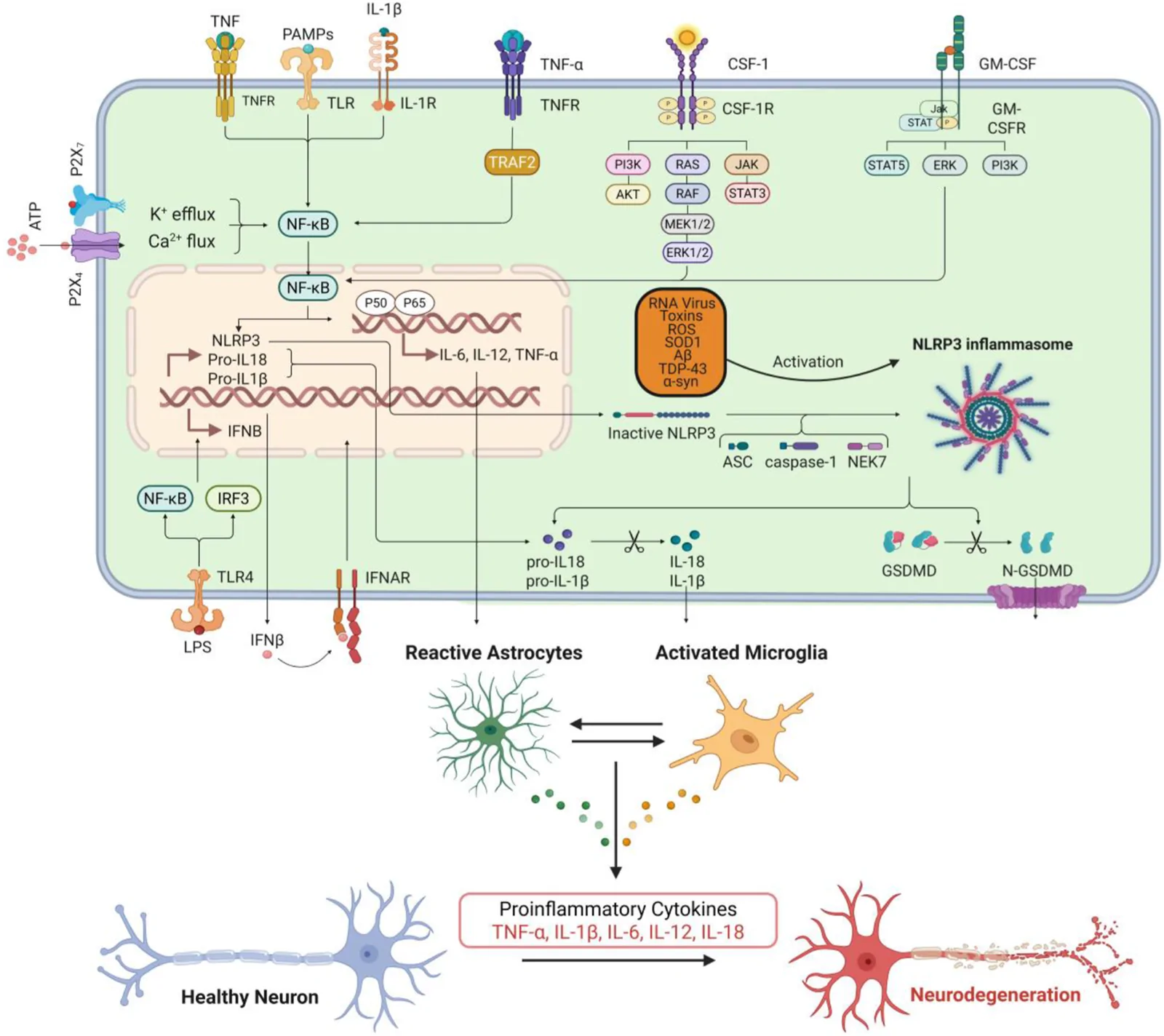
Biochemical process in the neuroinflammation involving microglia and astrocytes in the brain. Pathogen-associated molecular patterns, such as LPS and cytokines (TNF-α & IL-1β), bind to Toll-like receptors (TLRs) on the microglial surface, activating signalling pathways, such as NF-κB, MAPK, and PI3K/AKT. These pathways mediate the priming and assembly of the NLRP3 inflammasome via signals such as potassium efflux and calcium flux. The activated inflammasome complex, composed of NLRP3, ASC, and pro-caspase-1, cleaves pro-IL-1β and pro-IL-18 into their mature, proinflammatory forms and triggers gasdermin D (GSDMD)-mediated pyroptosis. The released inflammatory cytokines (TNF-α, IL-1β, IL-6, and IL-12) stimulate astrocytes to become reactive, releasing neurotoxic substances that contribute to the degeneration of healthy neurons. Other molecules implicated in neurodegenerative disease, such as RNA viruses, toxins, ROS, and misfolded proteins (e.g., SOD1, alpha-synuclein, TDP-43, and Aβ), are known to further oligomerize NLRP3, ASC, and procaspase-1, potentiating the inflammasome-induced inflammatory pathway.

As the primary immune cells of the CNS and major participants in neuroinflammation, microglial cells shift towards a proinflammatory phenotype in response to various stimuli (Afonina et al., 2017; Biswas, 2023). Whether initiated advertently or inadvertently, the immune response leads to the release of several inflammatory mediators, including TNF-α, IL-1β, and IL-6, which are major proinflammatory cytokines produced by activated microglia and astrocytes in the brain. Upregulation of these proinflammatory cytokines drives chronic neuroinflammation. Prolonged neuroinflammation can cause functional damage not only to neurons but also to astrocytes, microglia, oligodendrocytes, and other cell types. Neuroinflammation may also trigger systemic inflammation, which in turn can damage other organs. Therefore, early intervention to prevent the progression of neuroinflammation is the key treatment strategy to prevent irreversible structural and functional damage to the nervous system. Apart from steroidal drugs such as corticosteroids, several NSAIDs with different mechanisms of action have been in use for many years to treat neuroinflammation in neurological disorders. However, prolonged use of steroids and NSAIDs could lead to various health complications such as injury to the gastrointestinal tract, risk of cardiovascular diseases, hepatic and renal injuries, risk of intracerebral hemorrhage, etc (Bindu et al., 2020).

Several preclinical studies have demonstrated the beneficial effects of anti-inflammatory drugs, including natural bioactive compounds such as alkaloids, flavonoids, resveratrol, cannabidiols, ginsenosides, and curcumin as main or adjuvant treatments for controlling neuroinflammation in central nervous system diseases. The majority of these drugs are lipophilic and can cross the blood-brain barrier, protecting nervous tissue from neuroinflammation and oxidative stress via multiple mechanisms. These molecules could inhibit the formation and accumulation of abnormal proteins in nervous cells, prevent mitochondrial dysfunction, promote neurogenesis, and improve cognitive function in neurological disorders such as Parkinson’s disease, Alzheimer’s disease, Huntington’s disease, cerebral ischemia, multiple sclerosis, stroke, etc (Zhao et al., 2023). Bioactive compounds from plant sources, particularly dietary ones, are of paramount importance because they can target multiple pathways and help control neuroinflammation. In this review, phytochemicals from medicinal herbs and dietary supplements are shortlisted and discussed, drawing on abundant data to demonstrate their anti-inflammatory benefits in preclinical studies of neurological diseases.

A structured literature search was undertaken to identify published evidence on the anti-neuroinflammatory properties of phytochemicals derived from dietary and other naturally consumed sources. The literature search was conducted using PubMed, Scopus, Web of Science and Google Scholar. The search strategy incorporated keyword combinations related to several individual dietary phytochemicals, neuroinflammation, and their underlying molecular and cellular mechanisms. General search terms included phytochemicals, phytochemical names, dietary phytochemicals, bioactive compounds, natural compounds, neuroinflammation, neuroinflammatory response, microglia, astrocytes, inflammatory cytokines, interleukins, inflammasomes, oxidative stress, neuroprotection, etc. These terms were combined, as appropriate, with the names of phytochemicals using Boolean operators such as AND OR. Several search engines and keywords provided the same articles again in another search. Among them, original research articles that provided evidence on the anti-neuroinflammatory efficacy, neuroprotective properties, molecular targets and signaling mechanisms of dietary phytochemicals were considered for inclusion.

Evidence predominantly from preclinical studies, including from primary cell culture experiments and animal models, was considered to provide a broad assessment of the biological and translational relevance of the selected compounds. Relevant peer-reviewed reviews were screened to provide background information, identify important primary studies, contextualize the available evidence and identify additional relevant publications. Data from the following publications were excluded from the study: 1. Exclusive cell line studies, 2. Not peer reviewed, 3. Published in a language other than English, 4. Conference abstracts or inaccessible full texts, 5. Lacked sufficient methodological information. Also, when multiple publications reported similar findings, preference was given to studies that provided additional mechanistic evidence or greater relevance to neuroinflammatory conditions.

Following literature screening, phytochemicals were evaluated qualitatively based on the extent and consistency of available evidence, their anti-neuroinflammatory activity, mechanistic characterization, evidence across experimental models, and prospective therapeutic relevance. On this basis, ten phytochemicals were prioritized for detailed discussion: asiatic acid, asiaticoside, gingerols, glycyrrhizin/glycyrrhizic acid, mangiferin, piperine, punicalagin, shogaols, sulforaphane, and thymoquinone. These compounds were selected because a comparatively substantial body of literature supported their ability to modulate neuroinflammatory processes and provided mechanistic evidence relevant to their therapeutic utility. As we focused mostly on the abundance, several other phytochemicals were left out due to relatively less *in vivo* anti-neuroinflammatory data. Although resveratrol and curcumin possess well-established anti-neuroinflammatory activities, they were not included in the detailed analysis because their effects and mechanisms in neuroinflammation have been extensively addressed in recent reviews (dos Santos et al., 2024; Zhou and Hu, 2025). Their exclusion allowed the present review to focus on phytochemicals that have received comparatively less consolidated attention while showing a growing body of experimental evidence.

Given the narrative nature of the review, no formal meta-analysis or quantitative synthesis was undertaken. Instead, the retrieved evidence for the selected phytochemical was synthesized through a critical evaluation of the available evidence, using carefully constructed tables, with an emphasis on the experimental models employed and the effects on neuroinflammatory mediators, molecular targets, signaling pathways and functional benefits. Finally, detailed conclusions and future perspectives were provided.

## 2 Asiaticoside, asiatic acid, gingerols and shogaols


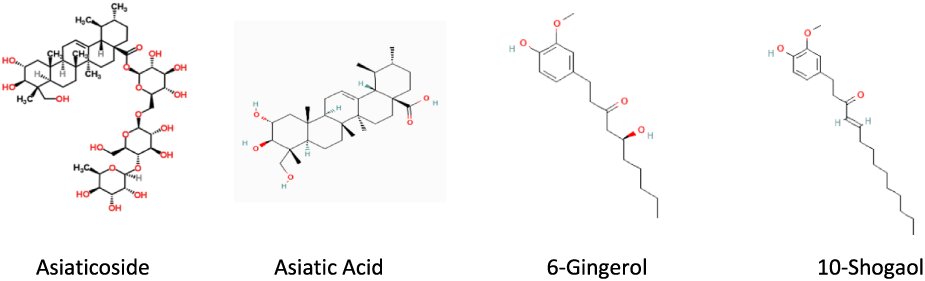


Asiaticoside and asiatic acid are rich in the dietary herb *Centella asiatica* L., a member of the *Apiaceae* family, and widely used in many traditional medicines and herbal preparations. Pharmacological effects of asiaticoside include anti-inflammatory, antioxidative, anxiolytic, antidepressant, wound-healing, neuroprotective, cardioprotective, hepatoprotective, and immunomodulatory activities (Yuan et al., 2024). Asiatic acid, an aglycone form of asiaticoside and a natural pentacyclic triterpenoid, exhibits hepatoprotective, cardioprotective, neuroprotective, anti-apoptotic, wound-healing, anti-inflammatory, anti-diabetic, and antimicrobial activities (Lv et al., 2018). Gingerols and shogaols are derived from ginger (*Zingiber officinale* Roscoe), a member of *the* *Zingiberaceae* family. These phytochemicals have been shown to have several health-promoting activities, including antioxidant, anti-inflammatory, antimicrobial, anticancer, neuroprotective, cardioprotective, anti-obesity, antidiabetic, anti-nausea, and antiemetic effects (Mao et al., 2019). Table 1 outlines the neuroprotective roles of asiaticoside, asiatic acid, gingerol and shogaol, primarily mediated by their anti-neuroinflammatory properties.

**TABLE 1:** Anti-neuroinflammatory activity of asiaticoside, asiatic acid, gingerol and shogaols in various neurological conditions.

Disease model	Drug dose/route	Molecular/biochemical/cellular findings	Functional benefits	Refs.
Asiaticoside				
Cerebral ischemia by BCCAO in mice	20, 40, 60 mg/kg/day; oral; 7 days	Inhibited the overexpression of IL-1β, IL-6, and TNF-α and phosphorylation of p38 MAPK in the hippocampus	Improved learning and memory	(Chen et al., 2014)
Spinal cord injury in the rat	30 mg/kg/day; i.p.; 7 days	Reduced NF-κBp65, TNF-α, IL-1β, IL-6 & p38-MAPK; Reduced oxidative stress; Reduced water content at the injury site	Reduced the spinal cord deficit scores	(Luo et al., 2015)
STZ-induced diabetic rats	20, 40 mg/kg/day; oral; 30 days	Reduced ROS production; Reduced the levels of TNF-α, IL-1β & NF-κB; Increased synapses in the hippocampus; Inhibited oxidative stress	Improved spatial learning and memory	(Yin et al., 2015)
Aβ-induced AD model of rats	5, 15, 45 mg/kg/day; oral; 7 days	Reduced the expression of IL-6 & TNF-α; Decreased caspases-3, and increased Bcl-2 in the hippocampus; Reduced Aβ deposition in the brain and hippocampal neurodegeneration	Improved learning and memory	(Zh et al., 2017)
Chronic unpredictable mild stress in the mice	20, 40 mg/kg/day; oral; 28 days	Increased the expression of 5-HT & NE; Reduced IL-1β, IL-6 & TNF-α in the hippocampus. Reduced the phosphorylation of NF-κBp65, expression of NLRP3 & caspase-1. Increased the level of cAMP & PKA	Improved cognitive function, reduced depression like behavior	(Wang et al., 2020a)
Aβ1-42 induced AD in mice	40 mg/kg/day; oral; 14 days	Reduced p38 MAPK, TNF-α, IL-β & IL-1 expression in the hippocampus; Increased PSD95; Reduced the number of IBA-1-positive cells	Mitigated cognitive dysfunction	(Liu et al., 2023)
MPTP-induced PD in mice	10, 60 mg/kg/day; oral; 15 days	Prevented loss of TH-positive cells; Reduced NLRP3 inflammasome, IL-1β in the substantia nigra	Ameliorated motor dysfunction	(He et al., 2024)
Intracerebral Hemorrhage in Zebrafish	600, 800, 1,000 μg/mL; Oral pool administration	Reduced IL-1β gene expression in the brain; Chelated iron accumulation in the brain. Reduced microglial activation and prevented neuronal apoptosis	Physical, behavioral and genotoxic observations showed no toxicity	(Srinivasan et al., 2024)
Asiatic acid				
D-galactose-induced aging in mice	5, 10, 20 mg/kg/day; 8 weeks	Reduced Bax, cleaved caspase-3, and retained Bcl-2 expression; Reduced IL-6, TNF-α, p-JNK & CD11b expression; Increased BDNF and reduced the GFAP expressions in the brain; Reduced oxidative stress	Not analyzed	(Chao et al., 2015)
Spinal cord injury in rats	30 or 75 mg/kg; orally/intragastrically after spinal-cord injury; once daily for 7 days	Suppressed myeloperoxidase activity; Reduced IL-1β, IL-18, IL-6, TNF-α & NLRP3 inflammasome; Increased NRF2/HO-1 levels, and downregulated the protein expression in SC; Reduced oxidative stress	Improved motor and sensory functions	(Jiang et al., 2016)
MPTP-induced mice	10 & 20 mg/kg daily oral for 14 days	Increased glutathione content and reduced ROS, NO, IL1β, IL-6, TNF-α, iNOS, COX-2, TLR4, TLR-2, & NF-κB p65& E2 in the striatum; Increased TH, BDNF, GDNF expression, reduced α-synuclein in the striatum	Not analyzed	(Chao et al., 2016)
AlCl_3_ induced AD in rats	30, 75 or 150 mg/kg/day orally for 6 weeks	Reduced AchE, amyloid-β1-42, β & γ secretases, GFAP & IBA-1, IL-1β, IL-6, IL-4, IL-2, TNF-γ, iNOS, NF-kβ, & COX-2 in the hippocampus and cortex	Attenuated memory and learning deficits	(Ahmad et al., 2018)
Intracerebroventricular injection of Aβ1-42 oligomer in AD mouse	Asiatic acid, 2.04 ± 0.16 mg/kg/day, intranasally, for 28 days	Reduced IL-1β, TNF-α, pTau-S369 & pTau-T231; Reduced oxidative stress	Improved learning and memory	(Islamie et al., 2023)
*Salmonella* typhimurium-infected mice	10 mg/kg/day, intragastrically, for 7 days before infection	Reduced the expression of IL-1β, TNF-α, IL-6, IFN-γ, IL-12β& NLRP3 inflammasomes in the hippocampus; Prevented the activation of microglia in the hippocampus	Prevented weight loss; Prevented anxiety and depression like behavior	(Zou et al., 2023)
Aβ1-42 induced AD in rats	75 mg/kg, i.p., for 28 days	Reduced the Aβ1–42, p-tau GSK-3β, NF-α and IL-6 levels in brain; Prevented aggregation of Aβ in the hippocampus and cortex; Reduced GFAP-positive cells in the hippocampus	Improved the spatial memory	(Halder et al., 2024)
6-gingerol				
LPS-induced rats	6-Gingerol 10 mg/kg/day, i.p., for 3 days before LPS	Inhibited GFAP and TNF-α protein levels in the brain	Improved spatial learning and memory	(Zhang et al., 2018)
EAE model in mice	6-Gingerol, 10 mg/kg/day, orally from the day of EAE induction through day 21	Suppressed the activation of NF-κB and MAPK signaling pathways; Reduced histopathological alterations in the spinal cord	Ameliorated the clinical symptoms of EAE	(Han et al., 2019)
MCAO in mice	5 mg/kg, intravenous; within 15 min of reperfusion	Inhibited the IL-1β, IL-6 & iNOS protein expression in the penumbra; Reduced the infarction area	Improved neurological function	(Liu et al., 2020)
Neonatal hypoxic-ischemic brain injury mouse model	Dose-ranging: 1, 2 & 4 mg/kg/day; ip	Reduced IL-1β, TNF-α, and IBA-1 in the brain Modified AKT, ERK, and NF-κB pathways and their epigenetic alteration; Reduced hypoxic area and neuronal death in the ipsilateral cerebral cortex and hippocampus	Improved short-term reflex performance; Corrected motor disability	(Zhao et al., 2021)
LPS-induced rats	20 or 50 mg/kg/day orally for 7 days	Reduced Il-1β, iNOS, APP, and β-secretase 1 and increased BDNF in the hippocampus and cerebral cortex	Ameliorated the learning and memory, locomotor and motor deficits	(Adetuyi and Farombi, 2021)
MCAO induced cerebral ischemia in rats	6 mg/kg 6-gingerol, i.p	Attenuated the NLRP3 inflammasome activation. Reduced the infarct area, neuronal cell necrosis and apoptosis in the brain. Enhanced autophagic activities	Improved neurological deficits	(Luo et al., 2021)
MCAO in rats	6-Gingerol: 5, 10, or 20 mg/kg; i.p; once daily for 7 days following MCAO.	Decreased the expression of COX-2 & IL-6 in the cerebral cortex and hippocampus; Reduced the infarct area in the brain and reduced the mitochondrial oxidative stress	Not analyzed	(Kongsui and Jittiwat, 2023)
LPS-induced depression model of rats	6-Gingerol: 50 mg/kg, orally, for 14 days	Reduced the expression of IBA-1, IL-1β, IL-18, NF-κB p65 and NLRP3 in prefrontal cortex; Reduced apoptosis in the prefrontal cortex	Reduced the depression like behavior	(Liu et al., 2024)
Traumatic peripheral nerve injury model (Sciatic nerve crush) of the rat	10 or 20 mg/kg; i.p.; once daily after sciatic nerve crush	Reduced IL-1β and TNF-α levels; Increased IL-4 & IL-10 in the injured tissue	Improved the locomotor sensory functions	(Al-Saedi et al., 2024)
6-shogaol				
MPTP induced mice	10 mg/kg/day; oral; 3-day pretreatment before the 5-day MPTP regimen	Reduced TNF-α, NO, iNOS & COX-2 proteins in the pars compacta of the substantia nigra and striatum; Increased TH-positive neurons in the substantia nigra- pars compacta and TH-IR fiber intensity in the striatum	Improved motor coordination reversed the bradykinesia	(Park et al., 2013)
AβO-injected AD mice model	10 mg/kg/day; oral; once daily for 5 days after intrahippocampal AβO_1_–_42_ injection	Reduced MAC-1 and GFAP-positive cells in the dentate gyrus.; Increased NGF levels and PSD-95 and synaptophysin in the hippocampus; Reduced the microgliosis and astrogliosis in the hippocampus	Improved memory	(Moon et al., 2014)
EAE-induced mouse model of MS.	6-Shogaol: 5 mg/kg/day, orally; 6-paradol: 5 mg/kg/day, orally; daily from days 29–42	Reduced the GFAP and IBA-1 positive cells and TNF-α expression in the spinal cord; Prevented demyelination and neuronal cell death in the spinal cord and gliosis	Improved EAE-relevant symptoms	(Sapkota et al., 2019)
TBI mice model	10, 20, or 30 mg/kg/day; i.p.; once daily for 14 days, starting 10 days after TBI	Inhibited the expression of NF-α, IL-1β and increased the level of BDNF in brain; Reduced oxidative stress, brain edema	Attenuated anxiety/depression-like behaviors	(Afzal et al., 2021)
*Proteus mirabilis* induced PD-like neurotoxicity in mice	10 mg/kg/day; oral; once daily for 22 days	Protected TH-positive cells in the striatum and substantia nigra pars compacta; Reduced IBA-1 positive cells	Ameliorated motor dysfunction	(Huh et al., 2023)
Arsenic, Lead, and Aluminium with HFD-fed mouse	Shogaol: 2 & 12 mg/kg; 72-day	Rescued the blood-brain barrier, and reduced the expression of TNF-α, IL-1β & GFAP genes in the hippocampus and cortex	Mitigated anxiety-like, depressive-like behavior and improved memory functions	(Ishaq et al., 2023)
Rotenone-induced PD in rats	10 or 20 mg/kg/day; oral for 28 days, with rotenone	Increased the levels of AChE, DA, GABA and normalizes the expression of IL-1β, TNF-α and NF-κB in the brain. Reduced the oxidative stress and neuronal damage in the brain	Improved the locomotive activity	(Rafeeq et al., 2024)
3-NP-induced Huntington’s disease in rats	10 or 20 mg/kg/day; oral; for 15 days	Improved the level of DA, 5-HT, NE, and GABA in the brain; Reduced IL-6, IL-1β, TNF-α, and NF-κB. & increased BDNF and NRF2 levels in brain	Improved the narrow beam walk and grip strength	(Jambi et al., 2024)
MPTP-induced PD mice model	6-Shogaol 20 mg/kg/day ± levodopa 80 mg/kg/day; oral; once daily for 26 days	Inhibited the expression of IBA-1, GFAP & TNF-α in the substantia nigra and striatum; Protected dopaminergic neurons in the substantia nigra. Reduced gliosis	Improved motor function, depressive-like behavior, olfactory function, and memory	(Kim et al., 2024)

Overall, asiaticoside, asiatic acid, gingerols and shogaols modulated neuroinflammation by reducing multiple proinflammatory molecules, including TNF-α, IL-1β, COX-2, iNOS, NO, NF-κB, TNF-γ, HMGB1, *etc.*, and by preventing nuclear translocation of NF-κB and HMGB1 and inhibiting NLRP3 activation. They also reduced the activation and number of IBA-1- and GFAP-positive cells in the brain, increased levels of brain growth factors, and modulated neurotransmitter levels, thereby highlighting their ability to modify multiple neuroinflammatory pathways and prevent inflammation-induced damage to CNS cells and their functions.

## 3 Glycyrrhizin/glycyrrhizic acid and mangiferin


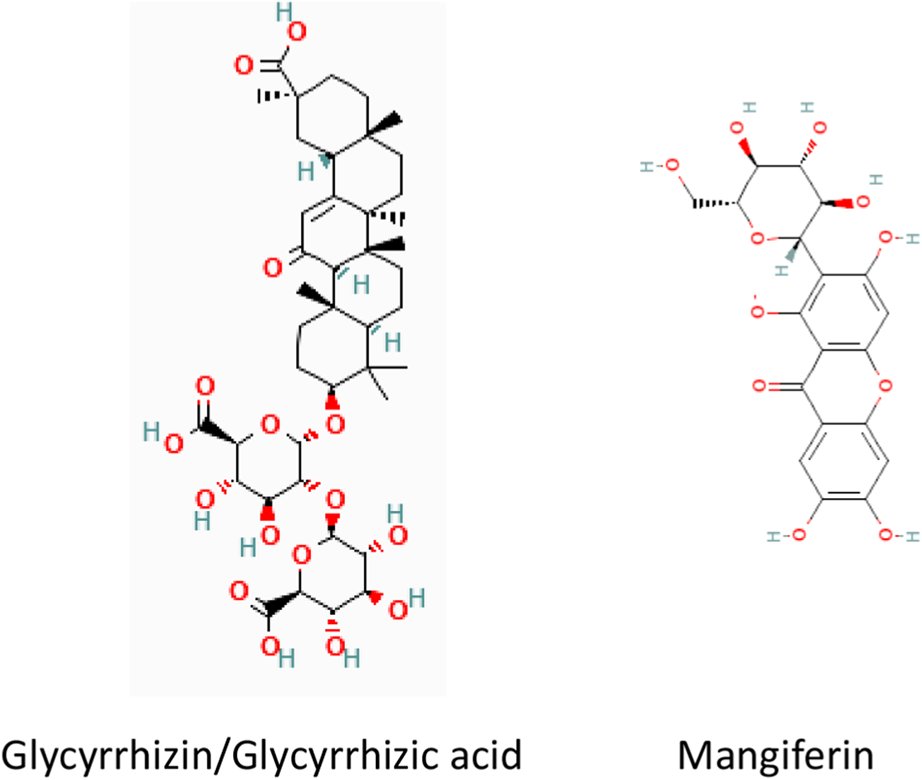


Glycyrrhizin/Glycyrrhizic acid is a triterpenoid saponin derived from *Glycyrrhiza glabra* L. (GB), which belongs to the *Leguminosae* family. Mangiferin is a phytochemical from *Mangifera indica* L., one of the most popular tropical fruits belonging to the *Anacardiaceae* family. Mangiferin, a natural xanthone polyphenolic glycoside, is the major bioactive constituent in its fruits. Mangiferin has been shown to have neuroprotective, gastroprotective, cardioprotective, nephroprotective, antidiabetic, anti-obesity, anticancer and lung- and skin-protective properties in several earlier studies (Shah et al., 2010; Zivković et al., 2024). The effect of glycyrrhizin/glycyrrhizic acid and mangiferin has been tested in several disease models, including cerebral ischemia, AD, HD, PD, neuroinflammation induced by LPS, conditions of depression, etc., as listed in Table 2.

**TABLE 2:** Anti-neuroinflammatory activity of Glycyrrhizin/Glycyrrhizic acid and mangiferin in various neurological conditions.

Disease model	Drug dose/route	Molecular/biochemical/cellular findings	Functional benefits	Refs.
Glycyrrhizin				
LPS-induced mouse model	10, 30 or 50 mg/kg/day; oral; for 3 days before LPS.	Reduced TNF-α & IL-1β in the brain Reduced COX-2, iNOS, IBA-1 & astrocyte activation in the hippocampus	Improved memory and learning	(Song et al., 2013)
Traumatic brain injury rat model	0.25, 1 or 4 mg/kg IV at 5 min post-TBI; long-term regimen: 4 mg/kg IV at 5 min and 6 h, then once daily through day 3; followed for 7 days	Glycyrrhizin binds to HMGB1 and prevented translocation of HMGB1 to neurons at injured sites Reduced the expression of TNF-α, IL-1β & IL-6 in injured sites	Improved motor and cognitive functions	(Okuma et al., 2014)
MCAO in rats	2, 4 or 10 mg/kg, single IV tail-vein injection 30 min after ischemia onset/30 min before reperfusion	Pretreatment inhibited the release of HMGB1 and cytochrome C & activity of Caspase 3 & serum levels, and mRNA expression of TNF-α, iNOS, IL-1β, and IL-6	Improved neurological deficits in locomotor function	(Gong et al., 2014)
MCAO-in mice	10 mg/mouse, i.p., at 1, 24 and 48 h after MCAO	Reduced IL-17 expression through HMGB1-TRL4 pathway; Suppressed the infrarct area	Improved neurological deficits	(Zhang et al., 2014)
Sevoflurane-surgery-induced recognition memory deficit in middle-aged mice	glycyrrhizin pretreatment 30 mg/kg, i.p	Peripheral IL-6 normalized by day 7; Hippocampal IL-6, IL-1β, TNF-α and glial activation persisted through day 20; Improved synaptic proteins; Suppressed TLR4/NFκB levels	Attenuated recognition memory deficits	(Yang et al., 2026)
MCAO in mice and rats	200 mg/kg/dose, i.p.; two injections—either immediately before MCAO plus 2 h after reperfusion, or at 0 and 3.5 h after reperfusion; 72-h endpoint	Reduced the expression of CD68, MPO, IFNγ and reduced the number of HMGB1-positive cells in the affected areas and in serum	Not analyzed	(Xiong et al., 2016)
Lithium/pilocarpine-induced status epilepticus in rats	50 mg/kg, i.p., single pretreatment 30 min before pilocarpine; outcomes at 3 and 24 h after SE.	Reduced the expression of IL-1β & TNF-α in the hippocampus and olfactory bulb; Reduced oxidative stress	Not analyzed	(González-Reyes et al., 2016)
Isoflurane-induced neonatal rats	20 mg/kg, i.p.; acute treatment; model exposure: 1.8% isoflurane for 4 h	Inhibited the expression of TNF-α & IL-1β, activation of HMGB1/NFκB pathway in the hippocampus. Prevented downregulation of synaptic proteins such as PSD-95 and SNAP-25	Improved the spatial memory	(Wang et al., 2016)
MPTP-induced PD in mice	16.8 or 50 mg/kg/day; i.p.; 21 days, initiated 30 min after the first MPTP dose	Reduced the expression and translocation of HMGB1 protein in TH-positive cells in the substantia nigra pars compacta. Neuroprotection to TH-positive cells and fibers in the substantia nigra	Not analyzed	(Santoro et al., 2016)
Postoperative cognitive dysfunction in mice	30 mg/kg/day; oral route; 3-day preoperative treatment	Reduced IL-1β, TNF-α & IL-6; Reduced NF-κB nuclear expression and reduced HMGB1 cytosolic expression. Reduced tau phosphorylation and Aβ40 and 42 concentrations in the hippocampus	Attenuated the severity of postoperative memory impairment	(Kong et al., 2017)
CFA induced inflammation in the mice spinal cord	5, 10, or 20 mg/kg/day; i.p.; 5 days, starting 1 h after CFA administration	Reduced CFA-induced expression of HMGB1, TLR4 and P65-NF-κb in the spinal cord	Alleviated the CFA-induced pain hypersensitivity	(Sun et al., 2018a)
EAE model in mice	Glycyrrhizin 10, 25, or 50 mg/kg, i.p., daily using three different schedules	Reduced GFAP & IBA-1 positive glial cells in the affected area of the spinal cord. Reduced HMGB1 expression in neurons and glia	Inhibited the progress of EAE.	(Sun et al., 2018b)
Subarachnoid hemorrhage model of the rat	15 mg/kg/injection; i.p.; immediately and at 6, 12, and 18 h after SAH induction	Reduced the number of HMGB1-positive cells; Reduced HMGB1 in the frontal cortex; Reduced TNF-α, IL-1β levels	Improved the neurological score	(Ieong et al., 2018)
Conditioned fear response after single prolonged stress (SPS) exposure in rats	Glycyrrhizin 0.05, 0.2, or 1 µg in 0.5 µL per hemisphere, into the basolateral amygdala before SPS exposure, followed by once-daily microinfusions for 6 days	Reduced the expression of HMGB1 and TLR4 in the basolateral amygdala; Inhibited the activation of microglia and astrocytes	Diminished the SPS-induced fear	(Lai et al., 2018)
Lithium-pilocarpine-induced status epilepticus in rats	Glycyrrhizin 50 mg/kg	Reduced HMGB1 levels in serum and hippocampus. Prevented translocation of HMGB1 from the nucleus to the cytoplasm in the hippocampus. Prevented neuronal damage in CA1, CA3 & Hilus of the hippocampus. Prevented BBB disruption	Reduced the mortality rate	(Li et al., 2019)
Spinal cord injury model of rats	Glycyrrhizin 50 mg/kg per dose, orally	Reduced NLRP3 inflammasome, HMGB1, IL-1b, IL-18, iNOS, TNF-α. Improved BDNF & GDNF levels. Reduced the Caspase-3 positive cells at the site of injury. Increased Arg-1 and IL-10 expression	Improved functional recovery	(Su et al., 2020)
PTZ induced seizures in adult zebrafish	Glycyrrhizin 25, 50, or 100 mg/kg; i.p	Inhibited the mRNA expression of HMGB1, TLR4, NF-κb, & TNF-α in the brain	Anticonvulsive effect and ameliorated seizure-related memory disruption	(Paudel et al., 2021)
Postoperative neuroinflammation in mice	Glycyrrhizin ammonium salt, 200 mg/kg, i.p	Reduced the expression of HMGB1 and TLR2 mRNA in cerebrum and hippocampus	Not provided for Glycyrrhizin	(Lin et al., 2021)
MPTP-induced zebrafish PD model	Glycyrrhizin: 8, 12, or 18 μM; co-exposure with 50 μM MPTP	Reduced the HMGB-1a, TLR-4b, NF-κb, IL-1β & IL-6 in the brain; Increased the length of DA neurons, reduced neuronal apoptosis; Prevented vasculature loss	Improved locomotor activity	(Ren et al., 2022)
Chronic hypoxia induced white matter injury model of mice	Glycyrrhizin 50 mg/kg, i.p., Daily for 7 days	Altered the HMGB1/TLR4/NFkB pathway in brain tissues. Inhibited the expression of HMGB1, IL1b, IL-6 & TNF-α in the brain; Promoted oligodendrocyte maturation; differentiation and myelin formation	Improved cognitive behavior	(Lou et al., 2025)
Glycyrrhizic acid				
Aβ1–42‐induced AD mouse model	Diammonium glycyrrhizinate 10 mg/kg/day, i.p. for 14 days	Inhibited the expression of COX‐2, iNOS, TNF‐α, IL‐1β, and IL‐10 mRNA & the relative protein levels of COX‐2 and iNOS in the hippocampus. Reduced the number of IBA-1-positive cells in the hippocampus	Attenuated the memory deficits	(Zhao et al., 2013)
Rotenone-induced PD rat model	Glycyrrhizic acid 50 mg/kg/day, i.p. for 4 weeks; administered 30 min before each rotenone dose	Reduced IL-1β, IL-6, TNF-α, COX-2 and iNOS levels in the midbrain; Reduced IBA-1 and GFAP-positive cells in the substantia nigra pars compacta	Not analyzed	(Ojha et al., 2016)
Diffuse axonal injury in rats	Glycyrrhizic acid 10 mg/kg, i.v. single dose; administered 30 min before induction of injury	Inhibited the protein expression of HMGB1 in the cerebrum. Inhibited the cytosolic release of HMGB1. Reduced TNF-α, MMP9, and IL-1β in the cerebral tissue	Prevented neurological deficits	(Pang et al., 2016)
Subarachnoid hemorrhage in rats	Glycyrrhizic acid 10 mg/kg, i.pt., once daily for 3 days	Inhibited the HMGB1 protein expression in the basilar artery. Inhibits the mRNA expression of IL-1β, IL-6, TNF-α, and IL-10 in the basilar artery	Improved the neurological functions. Reduced cerebral vasospasm	(Li et al., 2017)
LPS-induced mouse model	Glycyrrhizic acid 50 or 100 mg/kg/day, i.p. for 7 days	Reduced IL-1 β, TNF-α & TLR4 in the brain	Alleviated the LPS-induced memory loss and cognitive deficit	(Liu et al., 2019)
Hypoxic-ischemic encephalopathy in mice	Glycyrrhizic acid 16.8 mg/kg/day, i.p., for 14 days	Reduced IL-1β, IL-6, TNF-α & iNOS. Reduced STAT3 phosphorylation; Reduced the brain edema	Attenuated neurological behavior deficits	(Kartik et al., 2024)
MPTP-induced PD model of mice	Glycyrrhizic acid 50 mg/kg, i.p. once daily for 7 days	Reduced IL-1β, TNF-α, COX-2, and NF-κB in the striatum and substantia nigra; Reduced oxidative stress; Protected TH-positive dopaminergic neurons in the striatum	Improved locomotor function and behavior	(Guo et al., 2023)
5XFAD AD mice	Glycyrrhizic acid 150 mg/L in drinking water, *ad libitum*, from P56 to P100 (44 days)	Reduced pNFκB p65, RAGE & IBA-1 expression in the adult basal forebrain; Increased the expression of ChAT.	Not analyzed	(Fisher et al., 2025)
Mangiferin				
NP-induced HD in rats	Mangiferin 10 or 20 mg/kg/day, oral, for 14 days	Reduced TNF-α, IL-1β, and IL-6 levels	Improved cognitive function, motor coordination, and neurological scoring	(Lum et al., 2023)
SAMP8 accelerated-aging mice	Mangiferin 25, 50, or 100 mg/kg/day, oral gavage, for 4 weeks	Reduced IL-1β and TNF-α expression through modulation of the AMPK/mTOR/IRF5 pathway in the brain	Not provided	(Nong et al., 2025)
AlCl_3_-intoxicated AD mice	Mangiferin 20 or 40 mg/kg, oral, for the last 21 days of a 42-day study	Reduced oxidative stress, improved BDNF levels, reduced the AChE activity and reduced TNF-α and IL-1β in the hippocampus	Improved cognitive functions	(Kasbe et al., 2015)
LPS induction in mice	Mangiferin 50 mg/kg, oral, total 12 days	Reduced IL-6 and HO-1 levels in the hippocampus	Improved cognitive function	(Fu et al., 2015)
Doxorubicin induction in male SD rats	Mangiferin 30 or 60 mg/kg/day, oral, for 7 consecutive weeks	Dose-dependently reducing TNF-α mRNA and protein expression in the brain	Not analyzed	(Siswanto et al., 2016)
Postpartum depression in mice	Mangiferin 20 or 60 mg/kg/day, oral/intragastric	Reduced IBA-1, TNF-α, IL-6, and IL-1β in the brain; Reduced IBA-1 positive cells in the CA1 and dentate gyrus of the hippocampus	Reduced depression-like behavior	(Yan et al., 2022)
Hypoxic-ischemic encephalopathy in rats	Mangiferin 20 or 40 mg/kg, i.p. for 7 consecutive days	Reduced oxidative stress, reduced TNF-α, IL-1β, IL-6, and NF-κB p65 subunit expression. Attenuated cerebral infarct size, edema	Not analyzed	(Lv et al., 2016)
Subarachnoid haemorrhage in rats	Mangiferin 20 or 100 mg/kg, i.p., single administration after SAH induction	Reduced NF-κB p65 phosphorylation and decreased TNF-α, IL-6, NLRP3 inflammasome, IL-1, and IL-18 in the brain; Reduced brain edema	Reduced mortality, neurological deficits	(Wang et al., 2017)
Blast-induced TBI male SD rat model	Mangiferin 100 mg/kg, i.p., single dose; administered 20 min after blast injury	Reduced oxidative stress, reduced NLRP3 inflammasome, IL-1β and TNF-α levels in cerebrum; Reduced brain water content	Not analyzed	(Fan et al., 2017)
MCAO-induced brain injury in mice	Mangiferin 5 or 20 mg/kg/day, oral, for 7 days before MCAO	Reduced NLRP3 inflammasome, IL-1β & TNF-α levels in the cerebrum; Reduced histological alterations, brain edema and infarct size	Attenuated ischemia-induced neurological impairment	(Hao et al., 2023)
Chronic mild stress in mice	Mangiferin 20 or 40 mg/kg; intragastric; treatment for 3 weeks	Reduced NLRP3 inflammasome, IL-1β and IL-18 in the hippocampus	Reduced depression-like symptoms	(Cao et al., 2017)
ICV-quinolinic acid-induced cognitive deficits in rats	Mangiferin 20 or 40 mg/kg, orally administered for 21 days	Reduced the expression of TNF-α and IL-1β in the hippocampus and reduced the oxido-nitrosative stress, improved the cholinergic function and mitochondrial functions in the striatum and hippocampus	Improved motor activity and cognitive functions	(Arora et al., 2020)
6-OHDA-induced PD in rats	Mangiferin 45 μg; treatment from days 14–42 for 28 days	Reduced the oxidative stress, CoX-2 in the striatum; reduced TNF-α, IL-1β, IL-4, IL-6 levels and increased tyrosine hydroxylase in the brain	Improved behavioral and locomotor functions	(Tiwari et al., 2022)

Glycyrrhizin is known to act through the HMGB1-TLR4-NF-κB axis to bring its neuroprotective effects. In addition to preclinical studies, a randomized, placebo-controlled clinical trial found that glycyrrhizic acid, as an adjunctive treatment, reduced serum TNF-α levels and depressive symptoms in patients with high inflammation (Cao et al., 2020). Mangiferin also reduced neuroinflammation across multiple study models by reducing proinflammatory cytokines, improving antioxidative status and enhancing brain function.

## 4 Piperine and punicalagin


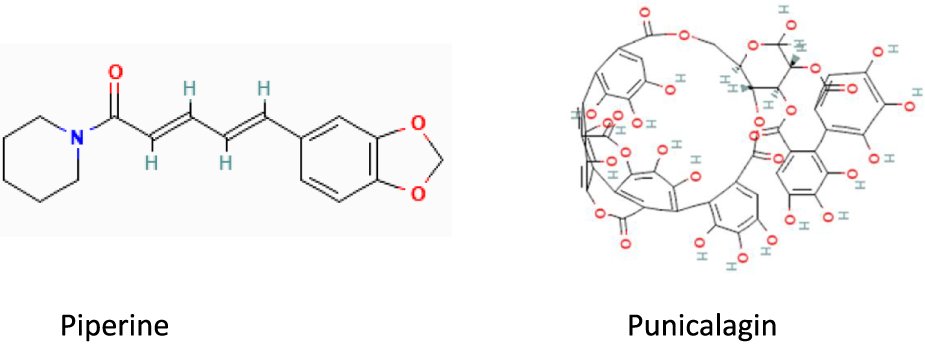


Piperine, the major alkaloid constituent of *Piper nigrum* L., has been extensively studied for its medicinal properties. Piperine stimulates appetite, increases saliva production, enhances intestinal motility, acts as an antitumor agent, increases pancreatic enzymatic activity, and prevents diarrhea. It also acts as an antioxidant, anticarcinogenic, anti-inflammatory, antiulcer, antithyroid, and antimicrobial agent (Shityakov et al., 2019). Piperine was studied for its efficacy against several animal models with a neuroinflammation component. Punicalagin is a key phytochemical from *Punica granatum* L. (Pomegranate), a member of the Punicaceae family. Punicalagin exhibited anticancer, antioxidant, hepatoprotective, antimicrobial, antiviral, angiogenic, anti-inflammatory, neuroprotective, gastroprotective, antidiabetic, and anti-hyperlipidemic activities (Rozadi et al., 2022).

In summary, both piperine and punicalagin exert their neuroprotective effects through anti-inflammatory activity, by reducing TNF-α, IL-1α, IL-1β, IL-6, COX-2, MCP-1, iNOS, NO, NF-κB, HMGB1, iNOS, TLR4, and increasing IL-4 and IL-10, prevented nuclear translocation of NF-κB and HMGB1, inhibited the activation of NLRP3, reduced the number of IBA-1- positive cells in the brain; by reducing oxidative stress; by increasing brain nutrients and balancing neurotransmitter levels (Table 3). The available evidence emphasizes the potential neurotherapeutic applications of piperine and punicalagin.

**TABLE 3:** Anti-neuroinflammatory activity of piperine and punicalagin in various neurological conditions.

Disease model	Drug dose/route	Molecular/biochemical/cellular findings	Functional benefits	Refs.
Piperine				
Rat lysolecithin-induced demyelination model	Piperine 5, 10 or 20 mg/kg/day; i.p. for 10 days, starting 3 days after LPC injection	Reduced TNF-α, IL-1β, NF-κB, and GFAP-positive glial cell expression at the injured site, increased IL-10 and FOXP3 expression, improved myelin repair	Improved spatial learning and memory	(Roshanbakhsh et al., 2020)
Autoimmune encephalomyelitis rat model	Piperine 5 mg/kg/day; i.p. from day 8 to day 29 post-immunization (22 days)	Reduced demyelination, reduced TNF-α, IL-1β and iNOS expression, increased IL-10, NRF2 and HO-1, Reduced MDA in the spinal cord	Alleviated neurological deficits and slowed the progression of EAE.	(Nasrnezhad et al., 2021)
Rat model of MCAO for ischemic stroke	Piperine 5 or 10 mg/kg; intragastric; for 14 consecutive days after ischemic stroke induction	Caspase-1, IL-1β, NLRP3, TLR4, and NF-κB p65 expression	Reduction in neurological deficits	(Yang et al., 2021)
Transient middle cerebral artery occlusion (tMCAO)-induced ischemic stroke in male Wistar rats	Piperine 10 mg/kg body weight/day; oral; 15 days	Suppressed mitochondrial dysfunction, reduced neuronal apoptosis, increased BDNF and CREB expression and reduced IL-1β expression in the penumbral frontal cortex; Reduced infarct volume	Improved neurobehavioral outcomes	(Kaushik et al., 2021)
Fluid percussion rat model of traumatic brain injury	Piperine 2.5 mg/kg; oral; once daily from Day 1 to Day 14	Reduced oxidative stress, reduced the levels of TNF-α, IL-1β, IL-6, improved the levels of DA, NE, 5-HT, GABA and the number of NRF2-positive cells in the cerebral cortex	Improved the neurobehavioral functions	(Kundu and Singh, 2023)
Streptozotocin-induced sporadic AD mice	Piperine 2.5, 5 or 10 mg/kg body weight; i.p. daily for 15 consecutive days, from Day 8 to Day 22 after ICV-STZ administration	Reduced IL-1β, IL-6, TNF-α and increased IL-4 and IL-10 in the mouse hippocampus	Improved locomotor activity and cognitive performance	(Wang et al., 2019)
D-galactose-induced senescent mouse model	Piperine 2.5, 5 or 10 mg/kg/day; i.p.; once daily for 28 days, beginning 14 days after the first D-galactose exposure	Reduced oxidative stress, TNF-α, IL-1β, and IL-6 levels and GSK-3β activation in the senescent mouse hippocampus	Improved cognitive impairment	(Wang et al., 2020b)
3-NP-induced HD rat model	Piperine 10 mg/kg; oral, twice daily for 4 consecutive days	Increased MAO activity, elevated serotonin levels, and reduced the number of GFAP-positive cells in the striatum	Mitigated locomotive behavioral deficits and anxiety-like behavior	(Salman et al., 2022)
6-hydroxydopamine-induced PD in mice	Piperine 10 mg/kg; oral; once daily for 15 days before 6-OHDA lesioning	Reduced apoptosis signaling proteins, TNF-α & IL-1β levels in the brain	Attenuated locomotor deficits; improved motor and balance activity	(Shrivastava et al., 2013)
Rotenone and iron-supplement induced PD in rats	Piperine 2.5 mg/kg/day; oral; for 28 days	Enhanced mitochondrial complexes I and IV activity, increased norepinephrine, dopamine, serotonin & GABA, reduced glutamate, TNF-α, IL-1β, and IL-6 in the striatum	Improved locomotor activity, grip strength, motor coordination, and balance	(Sharma et al., 2020)
Bilateral CCA occlusion model of PD dementia in mice	Piperine 10, 20 or 40 mg/kg; intragastric; once daily for 40 days	Reduced MAOB, β-secretase, AChE, Aβ42, TNF-α & IL-6 levels in the cortex	Improved behavioral functions	(Huang et al., 2022)
Punicalagin				
LPS-induced mice model	Punicalagin 1.5 mg/kg; oral, administered in drinking water; daily for 28 days	Reduced amylodogenic APP & BACE1 proteins, GFAP & IBA-1 positive cells in the hippocampus, IL-1β, IL-6, TNF-α, iNOS & COX-2 in the brain	Ameliorated the LPS-induced memory impairment	(Kim et al., 2017)
Methionine-induced hyperhomocysteinemia mice model	Punicalagin 1 mg/kg; i.p; daily for 30 days, concurrently with methionine	Reduced plasma homocysteine, TNF-α levels and reduced Aβ levels in plasma and the brain. Reduced oxidative stress, DNA damage and cell death in the brain	Prevented weight loss	(El-Missiry et al., 2018)
Collagenase type IV induced ICH in rats	Punicalagin 25, 50 or 75 mg/kg; oral; once daily for 15 days before ICH induction	Reduced NF-κB, IL-6, IL-18 & IL-1β levels; Prevented cell infiltration, cell damage and improved BBB integrity. Increased antioxidant activity	Not analyzed	(Zhang et al., 2020)
D-galactose -induced brain aging in mice	Punicalagin; 150 mg/kg, intragastric for 8 weeks	Reduced IL-18, IL-1β, TNF-α, IL-6, NLPR3 inflammasome, ASC, cleaved caspase-1, IL-1β, & IL-18 in the hippocampus	Prevented behavioral and cognitive decline	(Chen et al., 2023)
Manganese chloride induced PD in rats	Punicalagin 30 mg/kg/day; oral; once daily for 5 consecutive weeks	Increased NRF2 and HO-1 levels; Reduced NF-κB, TNF-α, IL-1β, IL-6, COX-2 and PGE2 in the brain; Reduced oxidative stress, neurodegeneration	Improved the motor functions	(Salem et al., 2023)
LPS-induced mice model	Punicalin 1,500 mg/kg/day; oral; once daily for 4 weeks before LPS administration	Reduced iNOS, COX2, IL-1β, IL-2, IL-6, TNF-α, TLR4, IRAK4, TRAF6, IKKβ, NF-κb p65, & HMGB1 in the brain. Prevented Aβ1−42 accumulation and reduced the IBA-1 and GFAP positive cells in the brain; Reduced oxidative stress	Improved memory and attenuated the anxiety- and depression-like behaviors	(Chen et al., 2024)
Manganese-induced PD in rats	Punicalagin 30 mg/kg; oral, by stomach gavage; once daily for 5 weeks	Reduced AChE activity and increased the levels of DA, NE, and 5-HT in the brain; Reduced TLR-4, GSK-3β, IL-Iβ, TNF-α, COX-2, NLRP3, NF-κb, PGE-2, Caspase-1 and Cysteine-aspartic protease-1 in the brain	Improved motor functions	(Abu-Elfotuh et al., 2024)
Spinal cord injury in the mouse	Punicalagin 50 mg/kg; i.p. once daily for 21 days or until euthanasia	Reduced microglial cells, reduced IBA-1 levels; Inhibited ferroptosis in the spinal cord	Promoted motor recovery	(Wang et al., 2025)
Neonatal hypoxic-ischemic encephalopathy model of the rat	Punicalagin 10 and 20 mg/kg; i.p., administered at 24-h intervals, beginning 24 h before hypoxia	Altered the AKT-FOXO4 signaling pathway to reduce autophagy. Reduced CCL6, CD74, etc. Reduced structural damage to the brain	Improved the learning and cognitive functions	(Shen et al., 2025)

## 5 Sulforaphane and thymoquinone


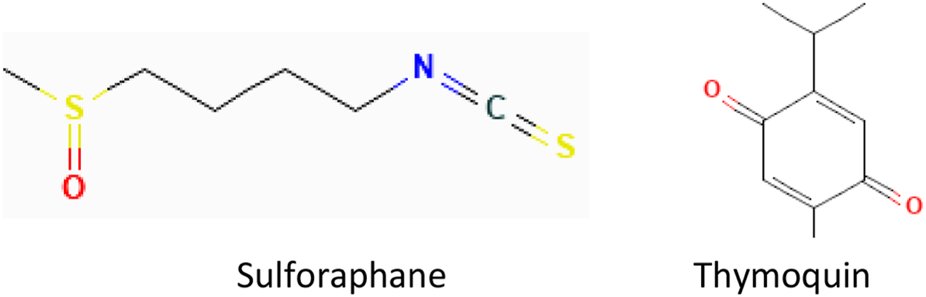


Sulforaphane (SFN) is an isothiocyanate stored in the form of glucoraphanin in many cruciferous vegetables such as broccoli, cauliflower, cabbage, kale, and others. In plants, glucoraphanin is converted to sulforaphane by the enzyme myrosinase. After being metabolized via the mercapturic acid pathway, SFN yields multiple metabolites. SFN is a dietary phytochemical with very low toxicity and antioxidant and anti-inflammatory properties (Vanducho et al., 2019). Thymoquinone (TQ) is one of the major constituents of *Nigella sativa* L. TQ has a wide range of therapeutic properties and is used to treat neurodegenerative, cardiac, respiratory, and urinary diseases. It possesses antioxidant, anti-inflammatory, anticancer, antibacterial, antimutagenic, and antigenotoxic activities (Farkhondeh et al., 2018).

Altogether, sulforaphane and thymoquinone, across various animal disease models, increased NRF2 activity, reduced neuroinflammation by decreasing IL-6, TNF-α, IL-1β, TLR-2, TLR-4, iNOS, COX-2, NF-κB, IRF-3, AChE activity, reduced IBA-1 and GFAP in glial cells, reduced neuroinflammation, neuronal degeneration, neuronal apoptosis, mitochondrial damage, and Aβ accumulation in the brain (Table 4). These pieces of evidence support the possibility of additional clinical trials for SFN and TQ, while some clinical trials for SFN in children with autism have already been completed.

**TABLE 4:** Anti-neuroinflammatory activity of sulforaphane and thymoquinone in various neurological conditions.

Disease model	Drug dose/route	Molecular/biochemical/cellular findings	Functional benefits	Refs.
Sulforaphane				
LPS-induced microglial cells from adult and aged mice	2.5 μM SFN; *in vitro*/*ex vivo*	Reduced oxidative stress, increased NRF2 DNA-binding activity, reduced IL-1β, IL-6 & NO levels	Not analyzed	(Townsend and Johnson, 2016)
Ethidium bromide induced MS -like pathology in rats	Sulforaphane: 1.5 or 3 mg/kg, i.p. once daily, day 8–35	Increased NRF2/HO-1/SIRT-1 levels; Reduced STAT-3, mTOR, and BACE-1 levels; Reduced demyelination and liver damage	Improved motor function, cognition	(Choudhary et al., 2026)
LPS-induced neuroinflammation in mice	5 mg/kg; i.p.; once daily for 5 consecutive days	Activated the AKT & elongation of microglial process; Reduced TNF-α & IL-1β; Increased IL-10	Improved behavioral functions	(Wu et al., 2019)
LPS-induced neurovascular inflammation in WT and Nrf2-KO mice	5 or 50 mg/kg, i.p., single dose, 24 h before LPS	Reduced IFN-γ, IL-6, MCP-1 & TNF-α; Increased IL-10 levels in both serum and brain; Improved neurovascular protection via both NRF2 independent and dependent pathways	Not analyzed	(Holloway et al., 2016)
LPS induced inflammation in mice	3, 10, 30 mg/kg, i.p., single dose, 30 min before LPS	Reduced serum level of TNF-α and increased IL-10; Enhanced dendritic branching and spine density, reduced MAC1 immunoreactivity in the CA1, CA3, and DG of the hippocampus	Reduced depressive-like behavior	(Zhang et al., 2017)
LPS-induced neuroinflammation in rats	1 mg/kg; i.p.; 5 days	Reduced TNF-α, IL-6, and IL-1β levels in both the brain and plasma; Reduced IBA-1 protein and NF-κB in the brain	Improved cognitive function	(Wang et al., 2020c)
LPS-induced recognition-memory impairment in Swiss mice	25 mg/kg/day; i.p.; 2 weeks	Decreased IL-6 and TNF-α; reduced oxidative stress in the prefrontal cortex; prevented neurodegeneration, Aβ accumulation, and microglial hyperactivity in this region	Improved recognition memory	(Alzahrani et al., 2024)
Aβ-oligomer-induced depressive-like behavior in SD rats	5 mg/kg/day, i.p., 7 days	Reduced serotonergic neuron damage in the dorsal raphe nucleus; Reduced oxidative stress; Reduced IL-1β & TNF-α levels in the brain	Reduced depression-like behavior; improved spatial learning	(Wang et al., 2020d)
Aβ1–42-induced AD rat model	10 and 20 mg/kg; Oral; 28 days, starting from day 7 post Aβ(1–42) administration	Reduced soluble Aβ(1–42) levels, prevented IRS-1 phosphorylation, reduced GSK-3β, TNF-α and NO levels and reduced AChE activity and oxidative stress in the hippocampus	Improved memory and learning	(Khan et al., 2024)
Hypothalamic astrocytes from aged rats	5 μM, 24 h, *in vitro*	Reduced IL-6 & COX-2 &oxidative stress; Increased IL-10, BDNF, GDNF, NGF, TrkA, TrkB, NRF2 & HO-1 & reduced AMPK, p21, & p65 NF-κB in the hypothalamic astrocytes	Not analyzed	(Santos et al., 2024)
Brain aging/neuroinflammation in rats	0.5 mg/kg, s.c., 5 days/week, 3 months	Reduced IFN-γ, IL-1α, IL-1β &TNF-α, & senescence markers in the cerebral cortex and hippocampus of adult male and female rats, but not in either sex of the older age groups	Improved memory in adult males and females, but not in older age groups	(Santín-Márquez et al., 2025)
Rat model of chronic hyperammonemia	0.5 mg/kg/day; i.p.; started after 2 weeks of ammonium diet and continued throughout the experiment	Reduced activation of astrocytes and microglia in the cerebellum. Enhanced polarization of microglia from M1 to M2; Reduced IL-1β & increased IL-4, IL-10, Arg1, and YM-1 levels in the cerebellum; Reduced extracellular GABA and normalized the membrane expression of GAT-3 in the cerebellum	Improved learning and motor functions	(Hernandez-Rabaza et al., 2016a)
Rat model of hyperammonemia	0.5 mg/kg/day; i.p.; started after 2 weeks of ammonium diet and continued throughout	Reduced microglial and astrocyte activation in the hippocampus and reduced IL-6 & IL-1β. Improved the expression of GABA, AMPA & NMDA receptors in the hippocampus	Improved spatial learning	(Hernández-Rabaza et al., 2016b)
Focal cerebral ischemia/reperfusion induced by right MCAO in rats	Sulforaphane 10, 20, 40 mg/kg/day; i.p.; 14 days before MCAO	Improved BBB function and decreased TNF-α, IL-1β, NO, iNOS, COX-2 and p-NF-κB p65 levels in the brain	Not analyzed	(Ma et al., 2015)
Cerebral ischemia/reperfusion model induced by MCAO in rats	Sulforaphane 5 or 10 mg/kg; i.p.; single dose after 60 min at reperfusion	Reduced the infarct volume, reduced infiltration of neutrophils to the ischemic site, reduced NLRP3 inflammasomes, IL-1β and IL-18 in the brain	Improved neurological scores	(Yu et al., 2017)
3-NP-induced Huntington’s disease in mice	2.5 or 5 mg/kg/day, i.p., daily, pretreatment beginning 30 min before 3-NP treatment	Reduced the lesion size, neuronal apoptosis, and microglial activation; Reduced TNF-α, IL-1β, IL-6, iNOS, and COX-2 in the striatum; Activated KEAP1, NRF2 in the striatum	Reduced the neurological score	(Jang and Cho, 2016)
PS1V97L transgenic mice AD model	5 mg/kg/day, i.p., 4 months	Reduced Aβ generation and aggregation, oxidative stress, and neuroinflammation in the brain	Improved cognitive function	(Hou et al., 2018)
MPTP induced PD in wild-type and NRF2-knockout mice	Sulforaphane 50 mg/kg; i.p.; two doses 8 h apart on day −1 followed by reinforcement doses on days 1, 3 and 5	Increased NRF2 levels in the basal ganglia, survival of dopaminergic neurons in the substantia nigra; Reduced astrogliosis, microgliosis, and the release of proinflammatory markers in the basal ganglia; Increased antioxidant enzymes	Not analyzed	(Jazwa et al., 2011)
Thymoquinone				
LPS-induced neuroinflammation in rats	TQ 2, 5, or 10 mg/kg; i.p.; 30 min before LPS; continued during behavioral testing	Reduced IL-6 and TNF-α in hippocampus; Reduced oxidative stress	Improved cognitive behavior	(Bargi et al., 2017)
LPS-induced neuroinflammation in mice	TQ 15 or 30 mg/kg; oral	Reduced TNF-α, IL-1β, amyloid-beta and β-secretase expression, AChE activity in the hippocampus and prefrontal cortex; Increased IL-10, ChAT and synaptophysin in these tissues	Improved cognitive function	(Adeoluwa et al., 2023)
D-galactose + AlCl3-induced AD rat model	TQ 20 mg/kg/day; intragastric; once daily for 14 days	Reduced oxidative stress, AChE activity, and TNF-α, IL-1β secretion; Increased antioxidant activity, BDNF, and Bcl-2 levels in the brain	Improved learning and memory	(Abulfadl et al., 2018)
AlCl3-induced neurotoxicity in rats	TQ 10 mg/kg i.p.; daily cotreatment for 6 weeks with AlCl3	Increased serotonin, dopamine, & acetylcholine; Reduced oxidative stress; Reduced TNF-α, IL-6 & increased BDNF levels in the brain	Improved cognitive behavior, reducing anxiety and depressive-like behavior	(Abbas et al., 2022)
D-galactose-induced aging/memory impairment in rats	TQ 2.5, 5, or 10 mg/kg; i.p.; 8 weeks	Reduced oxidative stress; reduced IL-1β & TNFα in hippocampus	Improved memory	(Oskouei et al., 2021)
Nonylphenol-induced neurotoxicity in male Wistar rats	TQ 2.5, 5, or 10 mg/kg; i.p	Reduced GFAP and α-syn in the brain; Reduced oxidative stress, rescued dentate gyrus, CA1, and CA3 histology	Improved learning and memory	(Lotfi et al., 2022)
3-NP-induced Huntington’s disease model in rats	TQ-loaded nanoparticles 10 or 20 mg/kg; oral; administered during 14-day 3-NP exposure	Reduced TNFα, IL-1β, IL-6, iNOS & COX-2 expression in the striatum; Reduced NF-κB phosphorylation and nuclear translocation; Rescued TH-positive cells in the striatum	Improved gait abnormalities and locomotor and exploratory behaviors	(Ramachandran and Thangarajan, 2018)
Electroshock-induced convulsions in rats	TQ 40 or 80 mg/kg; oral, daily for 14 days before induction	Reduced mTOR signaling, IL-1β, IL-6 & TNF-α in the hippocampus	Reduced convulsions	(Pottoo et al., 2021)
Lithium–pilocarpine-induced status epilepticus in male SD rats	TQ 10 mg/kg; i.p.; two pretreatment doses at 24 h and 1 h before pilocarpine	Reduced COX-2 & TNF-α in the cortex and hippocampus; NF-κB p65 in the hippocampus	Reduced seizures, improved cognitive function	(Shao et al., 2016)
Unpredictable chronic mild stress-induced depression-like behavior in mice	TQ 10 mg/kg; i.p.; daily from week 2–9 (8 weeks)	Reduced IL-1β, IL-6 & TNF-α & increased BDNF levels in the hippocampus and amygdala	Improved behavioral function	(Nazir et al., 2022)
Arsenic-induced hippocampal toxicity in Wistar rats	TQ 2.5 or 5 mg/kg/day; oral gavage; Pretreatment for 3 days, continued with arsenic up to day 11	Reduced oxidative stress, TNF-α & IFN-γ in the hippocampus	Improving neurobehavior	(Firdaus et al., 2018)
Aβ1-42-induced AD rat model	TQ 10 or 30 mg/kg, oral, for 7 days before and 10 days after Aβ1-42	Reduced TNF-α & IL-1β levels in the hippocampus; Reduced GFAP and IBA 1 activity	Reversed memory impairments	(Çam Öz et al., 2025)
Doxorubicin-induced neurotoxicity in rats	TQ10 mg/kg/day, i.p.; throughout experiment	Reduced oxidative stress, TNF-α, IL-17, HIF1-α, GRP78 & GADD153 in the cortex, medulla, and hippocampus	Not provided	(Kaymak et al., 2021)
Paclitaxel-induced peripheral neuropathy in mice	TQ 10, 20, 40 mg/kg/day for 28 days; oral gavage	Reduced TNF-α, IL-6 & IL-1β in DRG; Reduced TLR4/MyD88 and NF-κB/MAPKs inflammatory pathways in DRG	Reduced nociceptive hypersensitivity	(Xing et al., 2023)
Methotrexate (MTX)-induced neurotoxicity in male Wistar rats	TQ 10 mg/kg/day, p.o 21 days; MTX 20 mg/kg, i.p., single dose on Day 15	Reduced AChE activity; IL-1β, IL-6 & TNF-α in the brain; Reduced oxidative stress; Improved cerebral cortex, cerebellum, and hippocampus integrity	Not provided	(Behairy et al., 2024)
Acrylamide-induced cerebellar toxicity in rats	5 mg/kg/day; p.o. by gastric gavage; 14 days	Reduced oxidative stress, NRF2/NF-κB expression & DNA damage; Restored cerebellum histology	Not analyzed	(Famurewa et al., 2024)
Cypermethrin-induced neurotoxicity in rats	TQ: 5 and 10 mg/kg; Oral; 14 days	Reduced TNF-α, reduced activity of astrocyte and microglial cells; increased Bax, IL-10, AChE & NRF2 levels	Improved cognitive behavior	(Imam et al., 2024)
HFD-induced cerebellar neuronal injury in rats	TQ 300 mg/kg/day; oral; 4 weeks following 4 weeks of HFD	Reduced oxidative stress, serum levels of IL-1β, IL-6 & TNF-α, increased IL-10	Not analyzed	(Alrafiah, 2021)
Spinal cord ischemia–reperfusion injury in rats	TQ 10 mg/kg; i.p.; daily for 7 days before I/R induction and continued until euthanasia	Reduced oxidative stress, TNF-α & IL-1 levels and neuronal apoptosis	Improved locomotion function	(Gökce et al., 2016)
Cerebral ischemia–reperfusion-induced memory deficit in rats	TQ 10 mg/kg; i.p.; first dose immediately after cerebral I/R surgery, then daily for 14 days	Reduced oxidative stress and inflammation in the hippocampus, mediated through PPAR-γ	Improved memory and learning	(Hussien et al., 2020)
Sciatic nerve crush injury in rats	TQ 100 or 250 mg/kg/day; oral, once daily	Reduced NF-κB expression in Schwann cells and reduced MMP-9 & IL-10 levels in the injured tissue	Not provided	(Besin et al., 2022)
MCAO-induced focal cerebral ischemia/reperfusion in male rats	TQ-loaded niosome 5 mg/kg; IV; single injection within 2 h after stroke onset	Reduced oxidative stress, infarct volume, and edema in the brain; Reduced IL-6, IL-1β, TLR4 &TNF-α in the cerebral cortex, striatum	Improvement in reflex, sensory, and motor activities	(Hatami et al., 2023)
Cerebral ischemia/ischemia-reperfusion in mice	TQ 5 mg/kg, i.p.; once daily for 5 days before MCAO and once daily for 3 days after MCAO	Reduced the infarct volume and cerebral edema. administered brain; Microglial M2 polarization toward M2; reduced IBA-1, TNF-α & IL-1β increased TGF-β & IL-10 in the peri-infarct cortex	Improved sensorimotor function	(Zhao et al., 2024)
MPTP-induced PD model in mice	TQ 10 mg/kg, i.p.; daily for 7 days before MPTP and 7 days after MPTP	Reduced oxidative stress, IL-1β, IL-6, TNF-α, COX-2 & iNOS levels; Dopaminergic neuroprotection	Not analyzed	(Ardah et al., 2019)
EAE mouse model of multiple sclerosis	TQ 5 mg/kg, i.p.; daily administration	Reduced demyelination and reduced infiltration of mononuclear cells into the brain and spinal cord, reduced IFN-γ, IL-17, IL-6 & TNF-α; Increased IL-4, IL-10, and TGF-β in the lymph nodes and splenocytes	Reduced clinical severity	(Kazemi et al., 2024)

## 6 Conclusion and future perspectives

Treating chronic neuroinflammation at the earliest onset through medical interventions could help prevent the progression of CNS disorders into a stage of irreversible tissue damage. Since the continued use of anti-inflammatory drugs can cause multiple side effects, dietary herbs (Yamamura et al., 1995) or their phytochemicals, with proven efficacy in preclinical studies, could be explored for this purpose. However, the preclinical results should be interpreted with caution because differences in dose, route of administration, disease models, metabolism, and pharmacokinetic profiles may limit direct extrapolation to humans. In this review, asiatic acid, asiaticoside, gingerols, glycyrrhizin/glycyrrhizic acid, mangiferin, piperine, punicalagin, shogaols, sulforaphane and thymoquinone were shortlisted based on the availability of abundant data to demonstrate their prospective therapeutic utility. Some of these phytochemicals are already in clinical use or have undergone clinical trials for non-neurological diseases. For example, glycyrrhizin has been used clinically for liver disease for several decades in countries such as Japan (van Rossum et al., 1999); while gingerol (Ivashkin et al., 2022), mangiferin (Bourdas et al., 2024), punicalagin (Quirós-Fernández et al., 2019), sulforaphane (Ghannadi et al., 2025), and thymoquinone (Ali et al., 2021) have undergone clinical trials as an adjuvant or for pharmacokinetic studies.

### 6.1 Limited data from clinical trials

For neurological disorders, there are several clinical trials on crude extracts, standardized extracts and phytochemicals, with very few studies targeting neuroinflammation and providing direct evidence of their anti-neuroinflammatory activities. Published studies of asiatic acid, asiaticoside, gingerols, glycyrrhizin/glycyrrhizic acid, mangiferin, piperine, punicalagin, shogaols and thymoquinone as the sole therapeutic intervention for neurological disorders were not identified, except for pharmacokinetic studies. For sulforaphane, monotherapy clinical trials were undertaken, mostly for children with autism. Daily oral administration of sulforaphane (50–150 µmol) for an 18-week intervention period yielded statistically significant, reversible behavioral improvements in social interaction, aberrant behavior, and verbal communication, and was safe and well-tolerated. However, anti-neuroinflammatory or antioxidant effects were not studied in this trial (Singh et al., 2014). These behavioral findings were partially supported by a 15-week randomized, parallel, double-blind, placebo-controlled clinical trial in 57 children aged 3–12 years, with significantly reduced oxidative stress, as evidenced by increased GSH/GSSG ratio, and reduced neuroinflammation, as evidenced by reduced IL-6 and TNF-α expression (Zimmerman et al., 2021). Another recent randomized, double-blind, placebo-controlled, multi-center Trial in 108 children demonstrated that one-third of participants treated with sulforaphane showed at least a 30% decrease in clinician-rated scores by 12 weeks of treatment, while no significant change was observed in caregiver-rated scales (Ou et al., 2024).

Summary of outcomes identified from preclinical studies and clinical trials. Extensive preclinical and limited human studies indicate that these phytochemicals demonstrate significant anti-neuroinflammatory effects and prevent inflammatory damage to CNS cells. A few of the mechanisms by which these phytochemicals support brain function (Figure 2) under disease conditions include.

- Inhibiting neuroinflammation by reducing multiple proinflammatory molecules among TNF-α, IL-1α, IL-1β, IL-2, IL-4, IL-6, IL-12, IL-17α, IL-18; IFN- γ, COX-2, MCP-1, iNOS, NO, NF-κB, TNF-γ, HMGB1, GSK-3β, HIF-1α, iNOS, TLR2, TLR4, STAT3 etc.,
- Inhibited the phosphorylation of ERK & JAK, AKT/mTOR/STAT3 pathways; prevented nuclear translocation of NF-κB and HMGB1, inhibited the activation of NLRP3.
- Reduced the activation and number of IBA-1- and GFAP-positive cells in the brain.
- Increasing the levels of BDNF, GDNF, NGF, IL-10, etc
- Increasing the dopamine, GABA, norepinephrine levels and TH-positive cells in the brain
- Reduced ROS production and oxidative stress; increased DNA binding of NRF2; and activated antioxidant defenses (NRF2/HO-1), CREB, reduced HIF-1α, increased GSH/GSSG ratio, etc.

**FIGURE 2:**
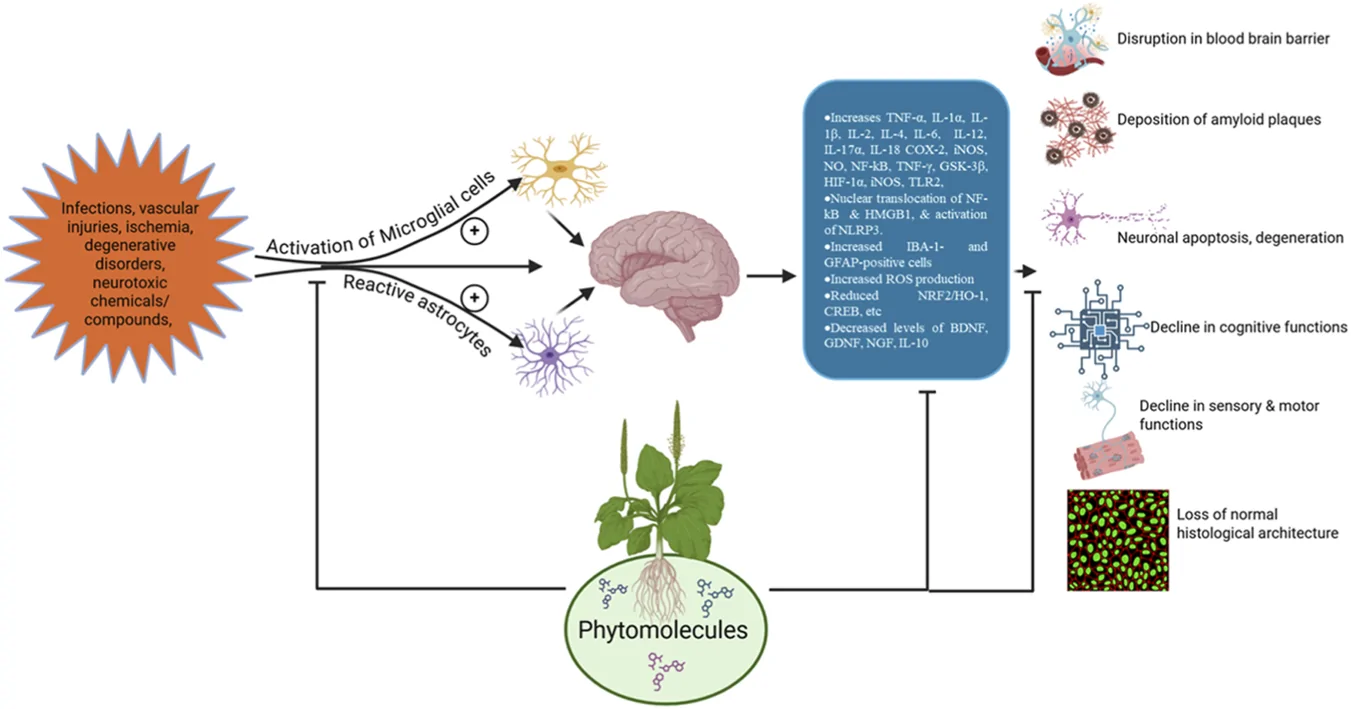
Role of phytochemicals in preventing neuroinflammation and their functional benefits.

Additionally, these phytochemicals possess strong antioxidant activity These two vital properties of these phytochemicals further inhibit the adverse consequences, such as cognitive impairment, motor function impairment, disruption in blood-brain barrier, transformation of inflammatory polarity in microglial cells, neuronal apoptosis, neuronal degeneration, neurotransmitter imbalance, disruption in the histological architecture of key areas of the brain such as hippocampus, prefrontal cortex, striatal region, cerebellum, etc.

A few mechanisms by which these phytochemicals support brain function under disease conditions include inhibiting neuroinflammation by reducing multiple proinflammatory mediators, inhibiting phosphorylation of key molecules, preventing nuclear translocation of NF-κB and HMGB1, and inhibiting NLRP3 activation, reducing the activation and number of IBA-1- and GFAP-positive cells in the brain, increasing the levels of brain growth factors, increasing anti-inflammatory molecules, modulating neurotransmitter levels in the brain, reduced ROS production and accumulation; increased DNA binding of NRF2; and activated antioxidant defenses (NRF2/HO-1), CREB, etc., reduced HIF-1α, etc.

### 6.2 Therapeutic challenges of using phytochemicals for neurological disorders

For clinical trials for neurological diseases, pharmacokinetic and pharmacodynamic data are essential for these phytochemicals. Among the molecules discussed, asiatic acid, gingerols, piperine, shogaols, sulforaphane, and thymoquinone are lipophilic, and their ability to cross the blood-brain barrier is higher, provided that multiple other structural and functional factors that control BBB passage are favorable. These phytochemicals face significant therapeutic limitations primarily due to extremely poor bioavailability, low water solubility, rapid hepatic metabolism, and rapid elimination, resulting in very low blood concentrations (Table 5) that fail to achieve their otherwise potent activity.

**TABLE 5:** The blood/serum/plasma half-lives of the phytochemicals.

Phytochemical	Route of administration	∼ half-life in blood (h)	Refs.
Asiaticoside	Intravenous and oral, in rodents	∼4	(Hengjumrut et al., 2018)
Asiatic acid	Intravenous, in rodents	∼2	(Lee et al., 2012)
Gingerols and shogaols	Oral, in humans	0.6–2.4	(Zick et al., 2008; Zhang et al., 2022)
Glycyrrhizin	Oral, in humans	2.7–4.8	(Yamamura et al., 1992)
Mangiferin	Oral, in humans	7.85 ± 1.72	(Hou et al., 2012)
Piperine	Oral, in rodents	<1.5	(Sahu et al., 2014)
Punicalagin	Oral, in rodents	5–9	(Chang et al., 2025)
Sulforaphane	Oral, in rodents	2.2	(Hu et al., 2004)
Thymoquinone	Oral, in rodents	∼2.5	(Ansar et al., 2020)

The reported half-lives of these phytochemicals vary significantly, and because the available data comes from different species, routes of administration and experimental settings, they cannot be directly compared. Based on the current data, while it is difficult to determine the effective daily dose, multiple daily doses may be needed to maintain therapeutically effective concentrations. Improving their pharmacokinetic properties, reducing systemic side effects, ensuring brain delivery in the required quantities, and maintaining biological activity for prolonged periods are essential for their translational applications.

Modifying physicochemical properties without compromising biological activity is an important approach, while nanotechnology-based drug delivery offers an attractive alternative to overcome several limitations of phytochemicals in their clinical utility. Encapsulation of phytochemicals within biodegradable liposomes, phytosomes, polymeric nanoparticles, solid lipid nanoparticles, nanostructured lipid carriers, nano-emulsions, and polymeric micelles can improve their solubility and stability, gastrointestinal absorptivity, protect them against rapid metabolism, enhance bioavailability, biological half-life, and crossing the BBB to enter the brain, and minimize side effects, maintaining efficacy (Islamie et al., 2023; Halder et al., 2024; Ramachandran and Thangarajan, 2018; Hatami et al., 2023; Ansar et al., 2020). Also, modulation of nanoparticle size, lipid composition, surface charge, and surface chemistry, as well as functionalization with BBB-targeting ligands, can further facilitate selective delivery to brain regions. For example, quantification of thymoquinone in the TQ-loaded mesoporous silica nanoparticles revealed enhanced delivery to the cortex, thalamus, hypothalamus and midbrain, reduced delivery to the cerebellum compared to free TQ (Fahmy et al., 2019). Also, piperine-loaded nanoparticles improved the dissolution and oral bioavailability. Oral piperine nanoparticles increased bioavailability 2.7-fold and produced approximately 16-fold higher brain concentrations at 10 h than unformulated piperine in PTZ-induced epileptic rats (Ren et al., 2019). Most evidence supporting brain-targeted nanophytochemicals remains preclinical, and concerns regarding long-term nanocarrier toxicity, biodistribution and accumulation, formulation reproducibility, scalability, standardization, and regulatory requirements continue to pose important barriers to clinical translation (Naim et al., 2026; Teja et al., 2022). Future research should prioritize strategies to overcome poor bioavailability and unfavorable pharmacokinetics and to increase therapeutic efficacy, rather than merely evaluating these phytochemicals in similar disease models or exploring new mechanisms of action.

Importantly, pharmacokinetic parameters may vary considerably between healthy individuals and patients with different disease conditions. For example, the reported half-life of glycyrrhizin is approximately 2.7–4.8 h in control subjects but increases to approximately 8–9 h during treatment in certain disease conditions (van Rossum et al., 1999). Thus, disease-specific pharmacokinetic studies are necessary to establish appropriate doses, dosing intervals, and formulations before these phytochemicals can be effectively translated into clinical practice. Also, co-administration of two potent phytochemicals not only enhances the bioavailability of the other but could also reduce activity in two distinct inflammatory pathways. However, some co-administration may adversely impact the bioavailability of the other. For example, the addition of glycyrrhizin reduced the bioavailability of asiatic acid (Guo et al., 2018). Thus, co-administration studies require detailed pharmacokinetic and pharmacodynamic data, as well as information on added toxicity, dose-ranging and timing, physical and chemical compatibility, disease specificity, and regulatory compliance for long-term use.

### 6.3 Limitations of available preclinical data for clinical translation

The major limitation of the available literature is that each study evaluated a different set of cytokines/interleukins and functional outcomes and lacked standardized preclinical investigations, making it difficult to synthesize and analyze their efficacy and adverse effects across doses (Tables 1, 2, 3, and 4). Also, throughout the review, it was observed that the same phytochemical affected different cytokines/interleukins across preclinical studies. While different models are expected to yield different outcomes, the observed differences mostly arise from the random set of parameters tested. Thus, a set of regulatory guidelines dedicated exclusively to demonstrating the reliable anti-inflammatory potency and efficacy of a phytochemical in preclinical studies will minimize variability across studies.

Also, while neuroinflammation is widely recognized as a core mechanism in the pathophysiology of neurological diseases, it is not generally used as a conventional, direct component of standard clinical grading scales for most neurological disorders. At the same time, it is measured as the most important factor in almost every preclinical study. Furthermore, clinical conditions are significantly affected by confounding factors, a feature uncommon in preclinical studies. It is doubtful whether preclinically efficacious phytochemicals function similarly or differently in humans across disease stages and severities. Also, the severity scales used in preclinical studies differ significantly from those used for clinical conditions. While phytochemicals may be efficacious in the early stages of the disease, they may become ineffective in mid- or advanced stages in controlling neuroinflammation. Also, if a phytochemical is efficacious in the early stages of one disease, it may not be similarly effective for other disorders at a similar stage, due to significant cellular and molecular heterogeneity, either systemically or within the disease microenvironment. It is not possible to identify the precise functional condition from preclinical studies. Also, the efficacy of phytochemicals on disease severity, specifically for the selected top-tier phytochemicals, needs to be evaluated in detail.

In preclinical studies, phytochemicals are generally considered safe, as these studies primarily focus on therapeutic efficacy or mechanisms of action. Additional evaluation is warranted when extrapolating these findings to humans, given interspecies differences and heterogeneity in experimental models, dosing regimens and dose responses across disease conditions. Also, the possible adverse effects of the selected top-tier phytochemicals need to be evaluated on long-term safety and potential drug interactions. Multiple conflicting reports on the tested phytochemicals, particularly regarding their targets and effects, warrant significant reconsideration. For example, while Glycyrrhizin is an active phytochemical considered safe in preclinical studies, high doses or long-term use may cause adverse effects in humans (Isbrucker and Burdock, 2006).

## 7 Summary

Neuroinflammation and oxidative stress are key mechanisms underlying many neurological disorders, and both are promising therapeutic targets. While current anti-inflammatory drugs pose significant systemic side effects, phytochemicals from dietary sources show promise as alternative treatments in preclinical studies. This review examined preclinical data on dietary phytochemicals for controlling neuroinflammation and discussed their limitations for clinical application. Despite encouraging preclinical evidence, further evaluation is warranted when extrapolating these findings to humans, given interspecies differences, heterogeneity in experimental models and dosing regimens, uncertainties in bioavailability and pharmacokinetics, and differences in outcome measures. Further studies are necessary due to the lack of regulatory guidelines for demonstrating the reliable anti-inflammatory potency and efficacy of a phytochemical in preclinical studies, which could compare their impacts on a scale. Prioritizing the selection of the best phytochemicals, or their combinations, with the highest bioavailability, optimal dosing, and proven long-term safety, while minimizing drug interactions and systemic toxicity through a standardized set of preclinical studies, is essential for advancing clinical trials and managing chronic neuroinflammation. It is also useful to understand how the chosen top-tier phytochemicals affect disease severity scales. Additionally, incorporating nanotechnology to improve bioavailability, target specificity, stability and therapeutic efficacy of top-tier phytochemicals or their combinations is being considered to enhance their clinical utility in controlling neuroinflammation in specific neurological disorders.

## Glossary

3-NP: 3-Nitropropionic Acid
5-HT: 5-Hydroxytryptamine (Serotonin)
6-OHDA: 6-Hydroxydopamine
AChE: Acetylcholinesterase
AD: Alzheimer’s Disease
AGE: Advanced Glycation End Products
AKT: AK strain transforming
**AlCl**_**3**_: Aluminium Chloride
AMPK: AMP-Activated Protein Kinase
APP: Amyloid Precursor Protein
Arg-1: Arginase-1
ASC: Apoptosis-Associated Speck-Like Protein Containing a CARD
Aβ: Amyloid-Beta
BACE1: Beta Amyloid Precursor Protein Cleaving Enzyme 1
BBB: Blood–Brain Barrier
BCCO: Bilateral Common Carotid Occlusion
BChE: Butyrylcholinesterase
BCL-2: B-Cell Lymphoma 2
BDNF: Brain-Derived Neurotrophic Factor
cAMP: Cyclic Adenosine Monophosphate
CAT: Catalase
CCL6: C-C Motif Chemokine Ligand 6
CD: Cluster of Differentiation
CFA: Complete Freund’s Adjuvant
ChAT: Choline Acetyltransferase
CNS: Central Nervous System
COX-2: Cyclooxygenase-2
CREB: cAMP Response Element-Binding Protein
DA: Dopamine
DOPA: 3,4-dihydroxyphenylalanine
DRG: Dorsal root ganglion
E2: 17β-Estradiol
EAE: Experimental Autoimmune Encephalomyelitis
ERK1/2: Extracellular Signal-Regulated Kinases 1 and 2
FOXO4: Forkhead Box O4
GABA: Gamma-Aminobutyric Acid
Gadd45b: Growth Arrest and DNA Damage-Inducible Beta
GADD153: Growth Arrest and DNA Damage-Inducible Protein 153
GDNF: Glial Cell Line-Derived Neurotrophic Factor
GFAP: Glial Fibrillary Acidic Protein
gp91phox: 91-kDa Glycoprotein Phagocyte Oxidase (NOX2)
GRP78: Glucose-Regulated Protein 78
GSDMD: Gasdermin D
GSH: Reduced Glutathione
GSK-3β: Glycogen Synthase Kinase-3 Beta
HD: Huntington’s Disease
HFD: High-Fat Diet
HIF-1α: Hypoxia-Inducible Factor-1 Alpha
HMGB1: High-Mobility Group Box one
HO-1: Heme Oxygenase-1
IBA-1: Ionized Calcium-Binding Adapter Molecule 1
ICH: Intracerebral Hemorrhage
ICV: Intracerebroventricular
IFN-γ: Interferon Gamma
IKKα/β-IκB: Kinase Alpha/Beta
IL: Interleukins
iNOS: Inducible Nitric Oxide Synthase
i.p.: Intraperitoneal
IRAK4: Interleukin-1 Receptor-Associated Kinase 4
IRF-3: Interferon Regulatory Factor 3
IRS-1: Insulin Receptor Substrate 1
LPS: Lipopolysaccharide
MAC-1: Macrophage-1 Antigen (CD11b/CD18)
MAPK: Mitogen-Activated Protein Kinase
MCAO: Middle Cerebral Artery Occlusion
MCP-1: Monocyte Chemoattractant Protein-1
MDA: Malondialdehyde
MMP-9: Matrix Metalloproteinase-9
MPTP: 1-Methyl-4-Phenyl-1,2,3,6-Tetrahydropyridine
mTOR: Mechanistic Target of Rapamycin
MyD88: Myeloid Differentiation Primary Response 88
NE: Norepinephrine (Noradrenaline)
NF-κB: Nuclear Factor Kappa B
NGF: Nerve Growth Factor
NLRP3: NLR Family Pyrin Domain Containing 3
NO: Nitric Oxide
Nrf2: Nuclear Factor Erythroid 2-Related Factor 2
NSAIDs: Nonsteroidal Anti-Inflammatory Drugs
p21: Cyclin-Dependent Kinase Inhibitor 1A (p21)
p47phox: 47-kDa Phagocyte Oxidase Protein
PD: Parkinson’s Disease
PGE2: Prostaglandin E2
p-JNK: Phosphorylated c-Jun N-Terminal Kinase
PKA: Protein Kinase A
PPAR-γ: peroxisome proliferator-activated receptor gamma
PSD-95: Postsynaptic Density Protein 95
pTau: Phosphorylated Tau
PTZ: Pentylenetetrazole
RAC1: Ras-Related C3 Botulinum Toxin Substrate 1
RAGE: Receptor for Advanced Glycation End Products
RNA: Ribonucleic Acid
ROS: Reactive Oxygen Species
SFN: Sulforaphane
SNAP-25: Synaptosomal-Associated Protein 25
SOD: Superoxide Dismutase
STAT3: Signal Transducer and Activator of Transcription 3
STZ: Streptozotocin
TBI: Traumatic Brain Injury
TDP-43: TAR DNA-Binding Protein 43
TH: Tyrosine Hydroxylase
TLR: Toll-Like Receptor
TNF-α: Tumour Necrosis Factor Alpha
TQ: Thymoquinone
TRAF6: TNF Receptor-Associated Factor 6
TrkA & TrkB: Tropomyosin Receptor Kinase A and Tropomyosin Receptor Kinase B
YM-1: Chitinase-3-Like Protein 3 (CHI3L3)
α-syn: Alpha-Synuclein
